# Neuronal apoptosis by HIV-1 Vpr: contribution of proinflammatory molecular networks from infected target cells

**DOI:** 10.1186/1742-2094-9-138

**Published:** 2012-06-22

**Authors:** Debjani Guha, Pruthvi Nagilla, Carrie Redinger, Alagarsamy Srinivasan, Gerald P Schatten, Velpandi Ayyavoo

**Affiliations:** 1 Department of Infectious Diseases & Microbiology, Graduate School of Public Health, University of Pittsburgh, 130 DeSoto Street, Pittsburgh, PA, 15261, USA; 2 Pittsburgh Development Center, Magee Womens Research Institute, 204 Craft Avenue, Pittsburgh, PA, 15213, USA; 3 NanoBio Diagnostics, 1196 Saint Andrews Lane, West Chester, PA, 19382-2312, USA

**Keywords:** HIV-1Vpr, Macrophages, Neuropathogenesis, Proinflammatory cytokines

## Abstract

**Background:**

Human immunodeficiency virus type 1 (HIV-1) induces neuronal dysfunction through host cellular factors and viral proteins including viral protein R (Vpr) released from infected macrophages/microglia. Vpr is important for infection of terminally differentiated cells such as macrophages. The objective of this study was to assess the effect of Vpr in the context of infectious virus particles on neuronal death through proinflammatory cytokines released from macrophages.

**Methods:**

Monocyte-derived macrophages (MDM) were infected with either HIV-1 wild type (HIV-1^wt^), Vpr deleted mutant (HIV-1∆Vpr) or mock. Cell lysates and culture supernatants from MDMs were analyzed for the expression and release of proinflammatory cytokines by quantitative reverse transcription-PCR and enzyme-linked immunosorbent assay respectively. Mitogen-activated protein kinases (MAPK) were analyzed in activated MDMs by western blots. Further, the effect of Vpr on neuronal apoptosis was examined using primary neurons exposed to culture supernatants from HIV-1^wt^, HIV-1∆Vpr or mock-infected MDMs by Annexin-V staining, MTT and Caspase - Glo® 3/7 assays. The role of interleukin (IL)-1β, IL-8 and tumor necrosis factor (TNF)-α on neuronal apoptosis was also evaluated in the presence or absence of neutralizing antibodies against these cytokines.

**Results:**

HIV-1∆Vpr-infected MDMs exhibited reduced infection over time and specifically a significant downregulation of IL-1β, IL-8 and TNF-α at the transcriptional and/or protein levels compared to HIV-1^wt^-infected cultures. This downregulation was due to impaired activation of p38 and stress-activated protein kinase (SAPK)/c-Jun N-terminal kinase (JNK) in HIV-1∆Vpr-infected MDMs. The association of SAPK/JNK and p38 to IL-1β and IL-8 production was confirmed by blocking MAPKs that prevented the elevation of IL-1β and IL-8 in HIV-1^wt^ more than in HIV-1∆Vpr-infected cultures. Supernatants from HIV-1∆Vpr-infected MDMs containing lower concentrations of IL-1β, IL-8 and TNF-α as well as viral proteins showed a reduced neurotoxicity compared to HIV-1^wt^-infected MDM supernatants. Reduction of neuronal death in the presence of anti-IL-1β and anti-IL-8 antibodies only in HIV-1^wt^-infected culture implies that the effect of Vpr on neuronal death is in part mediated through released proinflammatory factors.

**Conclusion:**

Collectively, these results demonstrate the ability of HIV-1∆Vpr to restrict neuronal apoptosis through dysregulation of multiple proinflammatory cytokines in the infected target cells either directly or indirectly by suppressing viral replication.

## Background

HIV-1 invades the central nervous system (CNS) during early infection via infiltrating monocytes and lymphocytes that are infected in the periphery [1-4]. Studies indicate that 40–50% of HIV-1 positive patients develop some form of HIV-1 associated neurocognitive disorders (HAND) [5-8]. Although productive HIV-1 infection of primary neurons has not been demonstrated, it is well accepted that neurons are affected by HIV-1 through indirect mechanisms. These include the release of proinflammatory cytokines/chemokines and viral proteins from HIV-1-infected target cells. The proinflammatory cytokines/chemokines and neurotoxins are released from infected and/or exposed monocytes/macrophages [9-11]. Thus, activation of macrophages appears to be crucial for the development of HAND.

Neuroinflammation is characterized by several proinflammatory events including the release of proinflammatory cytokines such as IL-1β, -6, TNF-α, and chemokines that drive this process [12]. IL-1β leads to NF-kB-dependent transcription of proinflammatory cytokines including TNF-α, IL-6 and interferon (IFN) [13]. TNF-α which functions through caspase-dependent cascade, is an important factor in various acute and chronic neurodegenerative disorders [14]. In the context of HIV-1-induced neuropathogenesis, higher levels of TNF-α, IL-1β, IL-6, IL-8, monocyte chemoattractant protein-1, macrophage inflammatory protein-1 and CXCL10 are observed *in vivo* and also in *in vitro* model systems [15-17]. In subjects with HAND, levels of these neuroinflammatory factors are associated with higher viral load in cerebrospinal fluid (CSF) [17-19]. In addition, HIV-1 gene products are also known to modulate the levels of these cytokines in macrophages. In *in vitro* systems utilizing macrophages as target cells, HIV-1 envelope protein gp120 has been shown to induce proinflammatory cytokines production through p38, MAPK and phosphatidylinositol 3-kinase (PI3K) pathways [16,20]. Tat also participates in HAND by stimulating cytokine/chemokine networks in monocytes and macrophages [21]. HIV-1-encoded viral protein R (Vpr) has recently been documented as having a substantial role in the development of neuropathogenesis [22-25]. Recombinant Vpr (rVpr) has been shown to modulate several chemokines at the transcriptional level by regulating NF-κB-mediated transcription [26,27]. It is important to note that several of these studies have been carried out using recombinant proteins at non-physiological concentrations. This has prompted us to consider studies utilizing relevant infectious HIV-1.

In this study, our goal was to evaluate whether Vpr deletion can reduce neuronal death in the presence of other neurotoxic viral proteins including gp120 and Tat. This also documents indirectly a role for Vpr on neuronal apoptosis in the presence of those viral proteins. Results indicate that absence of Vpr decreased MDM infection over time and that reduced the expression of selective proinflammatory cytokines IL-1β, IL-8 and TNF-α in MDMs at the transcript and/or protein levels. This reduction of proinflammatory cytokine production from MDMs makes the Vpr deleted virus less neurotoxic compared to its HIV-1 wild type (HIV-1^wt)^ counterpart.

## Materials and methods

### Reagents

HIV-1 YU2^wt^ and YU2∆Vpr plasmids were obtained from Dr. Serge Benichou, France. Neural progenitor (NP) cells were obtained from Millipore (Temecula, CA, USA), and human recombinant IL-1β, IL-8 and TNF-α as well as neutralizing antibodies against IL-1β, IL-8 and TNF-α were purchased from R&D Systems (Minneapolis, MN, USA). Extracellular signal-regulated kinase (ERK)1/2, p38 and JNK inhibitors (PD98059, SB203580 and SP600125, respectively) were purchased from Calbiochem (San Diego, CA, USA).

### Isolation and culture of MDMs

MDMs were generated from normal peripheral blood mononuclear cells (PBMC). Heparinized blood samples were purchased from Pittsburgh Blood Bank using appropriate Institutional Review Board approvals from University of Pittsburgh. PBMCs were isolated by Ficoll-Hypaque gradient centrifugation. CD14^+^ monocytes were purified by positive selection using anti-CD14 monoclonal antibody-coated magnetic microbeads (Miltenyi Biotech, Auburn, CA, USA) and cultured as described previously [28]. To obtain MDMs, CD14^+^ cells (0.5 × 10^6^ cells/ml) were cultured in DMEM (GIBCO, Gaithersburg, MD, USA) containing 10% fetal bovine serum 2 mM L-glutamine (Cambrex, Charles City, IA, USA) 1% penicillin-streptomycin (GIBCO, Gaithersburg, MD), 1 × 10^6^ IU/ml GM-CSF and 1 pg/ml M-CSF (R&D Systems, Minneapolis, MN, USA). Half the volume of media was replaced every third day with fresh media containing GM-CSF and M-CSF for 7–8 days to differentiate them into MDMs.

### Culture and differentiation of NP cells

NP cells at passage 2 to 6 were maintained in 35 mm plates coated with 20 μg/ml poly-L-ornithine and recoated with 5 μg/ml mouse laminin in ENStem-A neural expansion media (Millipore, Temecula, CA, USA) along with 0.5% penicillin-streptomycin, 2 mM freshly added L-glutamine and 20 ng/ml FGF-2. For neuronal differentiation NP cells were centrifuged at 1000 rpm for 3 minutes and the pellet was resuspended in ENStem-A neuronal differentiation media (Millipore, Temecula, CA, USA). The cell suspension was maintained in differentiation media in 8-well chamber slides for up to 2–3 weeks.

### Virus preparation and characterization

HEK293T cells (2 × 10^6^) were maintained in D-10 media and transfected with 5 μg of HIV-1 proviral constructs YU2^wt^ and YU2∆Vpr using polyjet reagent (SignaGen Laboratories, Gaithersburg, MD, USA) as per manufacturer’s instructions. Supernatants were collected 72 hours post-transfection, spun at 3000 rpm for 10 minutes and filtered through a 0.4 μm filter to remove cellular debris. All virus stocks were further concentrated by ultracentrifugation at 22,000 rpm for 1 hour at 4°C and were characterized for the presence of appropriate viral proteins by western blot using anti-Gag and anti-Vpr antibody (NIH, Germantown, MD, USA). Virus titer was measured by p24 enzyme-linked immunosorbent assay (ELISA) and the infectivity of the viruses was calculated by standard TZM-bl assay.

### Treatment of MDMs

Differentiated (7–8 day old) MDMs (2 × 10^6^ cells/well) were either infected with HIV-1^wt^ or Vpr deleted mutant (HIV-1∆Vpr) at a multiplicity of infection (MOI) of 0.1 for long-term infection or left untreated as a negative control. Infected and control MDMs were maintained for 21 days. Cell pellets and supernatants were collected every 24 hours up to day 4 and every 4 days from day 8 to day 20 to monitor virus replication and cytokine production. For assessment of MAPK signaling events, infected MDMs were activated on respective days with 1 μg/ml of lipopolysaccharide (LPS) for 4 hours. For analyzing cytokines in presence of MAPK inhibitors, MDMs were pretreated with 10 μM of SB203580 (p38 inhibitor), or SP600125 (JNK inhibitor) or PD98059 (ERK1/2 inhibitor) for 2 hours followed by infection with HIV-1^wt^ or HIV-1∆Vpr at an MOI of 0.1 or mock. Virus and mock-infected cultures treated with (dimethyl sulfoxide) were used as controls.

### Proinflammatory cytokine array profiling by quantitative reverse transcription-PCR

Post exposure or infection time points, MDMs were washed with cold phosphate-buffered saline (PBS) and total RNA was extracted using the RNeasy mini kit (Qiagen, Valencia CA, USA) according to the manufacturer’s protocol, with additional on-column DNase1 digestion (RNase-free DNase kit, Qiagen, CA, USA). RNA concentration was determined by spectrophotometry. The integrity of RNA was assessed by 260/280 ratio and analyzed by agarose gel electrophoresis. The RT² Profiler PCR Array (SABiosciences, Valencia, CA, USA) was used for mRNA profiling studies and the assay was performed according to the manufacturer’s protocol. Briefly, 1 μg of total RNA extracted from mock, HIV-1^wt^ or HIV-1∆Vpr virus-infected MDMs was converted to cDNA using a RNA first strand synthesis kit. The cDNA product was used to perform the gene expression array using a Taqman 7900HT machine. The data were normalized for endogenous controls (included in the array) and differential regulation of proinflammatory cytokines (fold change) was analyzed from the Ct values from day 0 to day 20 using SABiosciences web-based tools. Genes that are differentially regulated (+/− 2-fold) in infected cultures were determined.

### Measurement of cytokines by ELISA

Supernatants were collected at specific time points from MDMs exposed/infected with HIV-1^wt^ or HIV-1∆Vpr viruses and kept at −80°C. The concentration of TNF-α (BD Biosciences San Diego, CA, USA), IL-1β and IL-8 (R&D Systems, Minneapolis, MN, USA) were analyzed in supernatants by ELISA following the manufacturer’s protocol. The optical density (O.D) was determined for each well using ELISA plate reader and the concentration of the cytokines were calculated from the standard curve.

### Immunofluorescence staining

Neurons were differentiated on poly L-ornithine and mouse laminin coated 8-well chamber slides, fixed in 4% paraformaldehyde for 15 minutes, permeabilized with 0.1% Triton-X-PBS for 15 minutes. The cells were rehydrated by 3 washes of PBS and 5 washes of 0.5% bovine serum albumin (BSA). After blocking with 2% BSA for 1 hour the neurons were incubated with primary antibodies against microtubule-associated protein 2 (MAP2) (1:250), β-III tubulin (1:300) and glial fibrillary acidic protein (GFAP) (1: 500) overnight at 4°C. The cells were washed 5 times with 0.5% BSA and were further incubated with Alexa Flour 488 goat anti-mouse-IgG, anti-rabbit-Cy3. After 5 washes with 0.5% BSA and 5 times with PBS the nuclei were stained with Hoechst 33342 (1 μg/ml) for 30 seconds. The slides were mounted and staining was checked under microscope.

### Treatment of neurons with specific neutralizing antibodies against proinflammatory cytokines

Primary neurons (3 × 0^4^ cells/well) were exposed to supernatants from HIV-1^wt^, HIV-1∆Vpr and mock-infected MDMs (10% of the volume of the total media) collected on day 8 or day 12 as well as recombinant IL-1β (10 ng/ml), IL-8 (10 ng/ml) and TNF-α (100 ng/ml). The cells were replenished with media containing neutralizing antibodies against IL-1β (5 μg/ml), IL-8 (5 μg/ml) and TNF-α (10 μg/ml) (R&D Systems, Minneapolis, MN, USA). The neuronal apoptosis was measured 24–48 hours after infection.

### Annexin-V FITC staining

Analysis of apoptosis was carried out using the apoptosis detection kit (BD Biosciences, San Diego, CA, USA) as per the manufacturer’s instructions. Briefly, neurons were exposed to HIV-1^wt^, HIV-1∆Vpr, mock-infected MDM supernatants (10% v/v) and recombinant cytokines for 24–48 hours. To detect apoptosis, infected media were removed, the cells were washed twice with 1X PBS and then once with 1X Annexin V binding buffer. Neurons were stained with Annexin V-FITC diluted (1:10) in 1X binding buffer for 15 minutes at room temperature in the dark, then washed once with 1X binding buffer. The apoptotic neurons (%) were quantified by nuclei staining with Hoechst 33342 and analyzed by microscopy. Neuronal apoptosis was calculated from the percentage of cells stained with Annexin-V.

### MTT assay

The effect of HIV-1Vpr-mediated proinflammatory factors on inducing neuronal death was also assessed using MTT assay. Briefly, 1 × 10^5^ primary neurons in each well of a 96-well plate (in triplicate) were exposed with the supernatants from mock, HIV-1^wt^ and HIV-1∆Vpr-infected MDMs (10% v/v) as well as recombinant cytokines and incubated at 37°C in 5% CO_2_ for 24 hours. The neurons were incubated with 1.2 mM of MTT solution for 4 hours at 37°C_._ The formazan crystals were solubilized in 100 μl DMSO and by shaking the plate for 10 minutes. The absorbance was measured at 540 nm. Mock-infected cells produced the highest O.D reflecting the highest cell survival and cells treated with DMSO were used as a negative control.

### Caspase - Glo® 3/7 assay

Caspase-3/7 activities were measured using Caspase-Glo® 3/7 Assay kit (Promega, Madison, WI, USA) according to manufacturer’s instructions. Briefly, 5 × 10^4^ primary neurons differentiated in each well of a 96-well plate were exposed to supernatants of mock, HIV-1^wt^ and HIV-1∆Vpr-infected MDMs (10% v/v) for 24–48 hours. The cells were incubated for 1 hour at 37°C with equal volume of Caspase-Glo® 3/7 reagent to the volume of culture medium. The luminescence that is proportional to caspase 3/7 activities was determined by luminometer. A negative control consisting of cells without MDM supernatant treatment was also included in each assay.

### Western blot

MDMs were washed twice with cold PBS and lysed in RIPA buffer containing 50 mM Tris (pH 7.5), 150 mM NaCl, 1% Triton-X100, 1 mM sodium orthovanadate, 10 mM sodium fluoride, 1.0 mM phenylmethyl-sulfonylfluoride, 0.05% deoxycholate, 10% sodium dodecyl sulfate (SDS), 0.07 trypsin inhibitor units/ml aprotinin, and protease inhibitors Leupeptin, Chymostatin, and Pepstatin (1 μg/ml; Sigma). Cell lysates were clarified by centrifugation and total cell lysates (50 μg) were separated on a SDS-PAGE gel, transferred, and the expression of proteins were detected with anti-ERK1/2, anti-p-38 (total and active; Cell Signaling Technology, Beverly, CA, USA), anti- SAPK/JNK (Cell Signaling Technology, Beverly, CA, USA), and anti-poly ADP-ribose polymerases (PARP; total and cleaved; Cell Signaling, Beverly, CA, USA). Anti-tubulin (NeoMarkers, Fremont, CA, USA) was used as loading control. Blots were developed using ECL kit (Pierce, Richmond, Illinois, USA). The results were analyzed and densitometric measurements were normalized against total proteins or α-tubulin expression levels.

### Statistical analysis

Results were expressed as mean ± SEM for three experiments. The data were analyzed using student’s *t*-test for normally distributed data with equal variances and *P* < 0.05 was considered significant. The immunoblotting images were quantified using Image J software. For qRT-PCR data analysis SABiosciences web-based software was used. Promoter analysis was performed using Genome TraFac software (http://www.ncbi.nlm.nih.gov/pubmed/17178752?dopt=AbstractPlus) as described [29].

## Results

### Characterization of viruses and replication kinetics of HIV-1^wt^ and HIV-1∆vpr in MDMs

**Figure 1:**
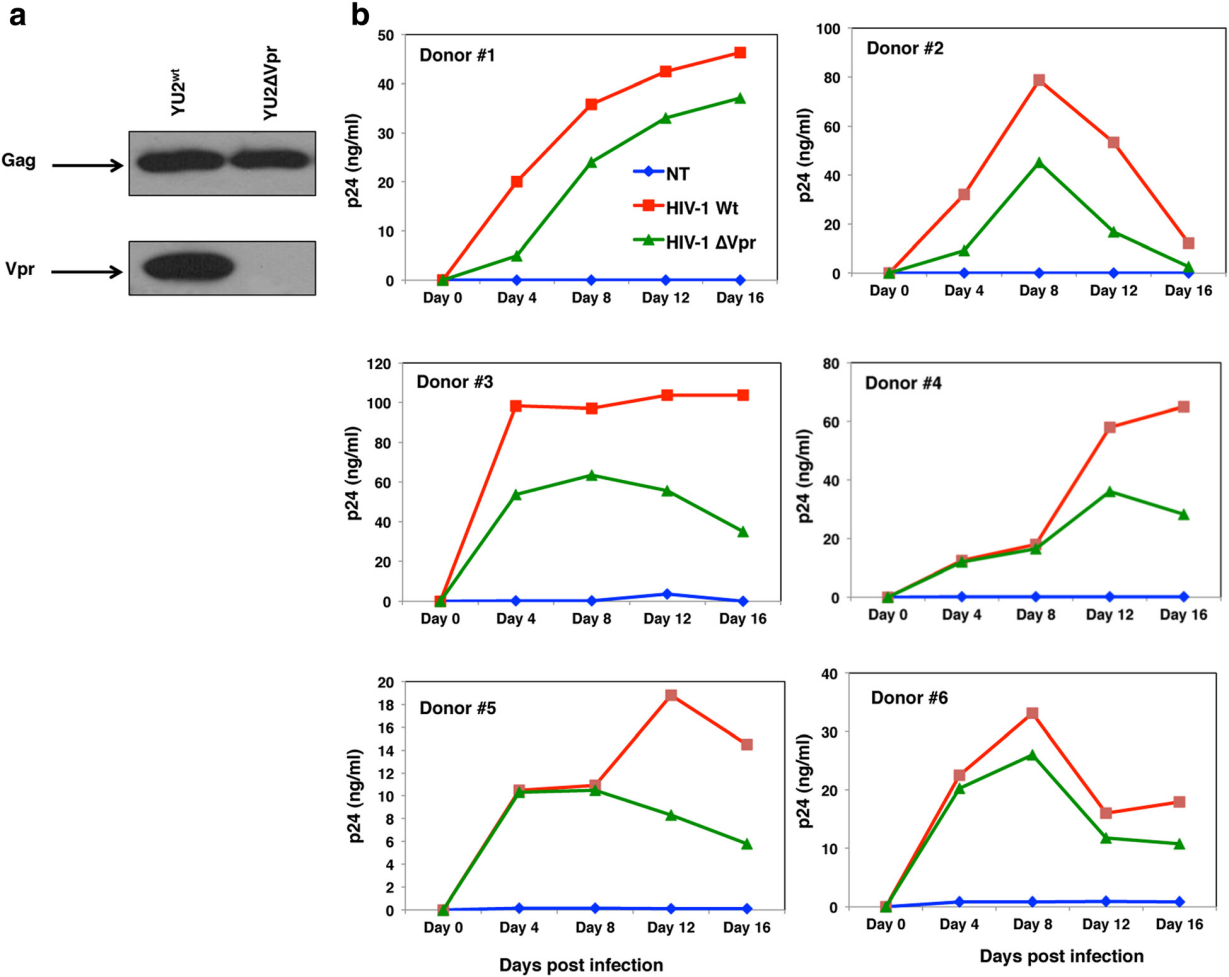
**Characterization and kinetics of HIV-1**^**wt**^**and HIV-1∆Vpr virus particles. (a)** HEK 293 T cells were transfected with 5 μg of HIV-1 YU2^wt^ and HIV-1 YU2∆Vpr plasmids. Viruses collected after 72 hours were filtered, concentrated by ultracentrifugation and western blot analysis was performed for Gag and Vpr. **(b)** Productive infection of MDMs by viruses. MDMs were infected with HIV-1^wt^ and HIV-1∆Vpr viruses at an MOI of 0.1 or left untreated as negative control (NT). The release of virus particles from infected MDM culture was monitored by p24 ELISA using the supernatants on days as indicated. The data are representatives of one of six independent experiments.

HIV-1 YU2^wt^ and YU2∆Vpr were produced through transfection of respective proviral DNAs into HEK293T cells. Supernatants were collected and virus particles in the culture supernatants were characterized for the presence of p24 and Vpr by western blot using specific antibodies (Figure 1A). Comparable expression level of Gag-p24 was found in both HIV-1^wt^ and HIV-1∆Vpr viruses, whereas, presence of Vpr was observed only in HIV-1^wt^ as expected.

For virus replication studies, MDMs from multiple normal healthy donors (N = 6) were infected with an equal amount of HIV-1^wt^ or HIV-1∆Vpr according to standard protocols described in Methods. To assess virus replication kinetics, supernatants at different time points were collected and virus titer was measured by p24 ELISA (Figure 1B). Results indicate that viral infection increased with time in all donors. Interestingly, removal of Vpr suppressed but not completely abolished HIV-1 replication in MDMs over time and this pattern is consistent in all tested donors suggesting that HIV-1 Vpr plays a significant role in MDM infection although it is not absolutely essential for infection.

### Differential regulation of proinflammatory factors in HIV-1^wt^, HIV-1∆vpr and mock-infected MDMs

It has been suggested that HIV-1-infected macrophages/microglia-mediated neuronal dysfunction are in part via proinflammatory factors/neurotoxins released by these cells [30]. Several viral proteins including Env, Nef, Tat and Vpr have been implicated in inducing proinflammatory responses in macrophages [31]. To better understand whether absence of Vpr causes any significant difference in proinflammatory cytokine expression in MDMs, we used normal donor derived MDMs infected with HIV-1^wt^ or HIV-1∆Vpr or mock for focused qRT-PCR array. Compared to mock/control MDMs, HIV-1^wt^-infected cells showed an enhanced expression of a number of cytokines and proinflammatory genes at different time points (Table 1). Among the upregulated genes, IL-1β and IL-8 exhibited a higher fold increase over mock treated, whereas, TNF-α, IL-22, IL-10 and C3 showed a modest increase in multiple donors (Table 1). During the infection phase (5–20 days post infection) IL-1β, IL-8 and C3 remained at a high level, whereas, other proinflammatory factors did not show any difference between infected and uninfected controls.

**Table 1:** **Differentially regulated inflammatory genes in MDMs infected with HIV-1**^**wt**^**compared to mock/uninfected (N = 6)**

**Genes**	**Day1**	**Day2**	**Day3**	**Day4**	**Day8**	**Day12**	**Day16**	**Day20**
CD40LG	-	-	-	-	2.00	2.32	-	3.02
IFNA2	−2.70	-	−2.25	-	-	-	−2.04	-
IL10	3.596	-	-	-	-	-	-	3.224
IL13	-	-	-	-	-	-	2.215	-
IL1A	9.54	5.83	-	-	-	2.63	-	-
**IL1B**	**55.89**	**31.59**	**3.58**	**2.31**	**2.19**	**7.97**	**8.09**	**9.87**
IL1F5	-	-	-	−3.40	-	2.135	-	−4.52
IL1F6	-	−2.015	−2.94	-	-	-	-	−2.38
IL1F8	-	−2.42	-	−4.49	-	-	-	−18.56
IL1F9	12.81	4.194	-	-	3.76	-	-	2.54
IL22	2.237	-	−3.18	−2.12	-	-	-	2.334
IL5		-	−2.13	-	2.31	5.41	2.81	-
**IL8**	**20.63**	**11.56**	**3.146**	**2.16**	**2.08**	**3.22**	**3.88**	**6.698**
IL9	−2.46	−3.445	-	-	-	2.02	-	−7.88
LTA	-	-	-	-	4.74	3.19		-
SPP1	-	-	-	−2.26	−2.21	−2.77		−2.43
TNF	3.13	-	-	−2.69	-	-	-	2.20
IL10RA	-	2.03	-	-	-	-	-	-
IL9R	-	-	−2.27	-	-	-	-	-
IL5RA	-	-	−4.33	-	-	-	-	-
BCL6	3.31	2.50	-	-	-	-	2.01	3.185
C3	8.49	7.86	3.35	2.52	3.16	2.03	-	8.90
C4A	-	-	-	-	3.49	-	-	2.35
CEBPB	-	-	-	-	-	-	-	3.59
CRP	-	-	-	-	-	-	−2.27	-
IL1R1	-	- 2.14	-	-	-	-	-	-
IL1RN	-	−2.02	−2.97	-	−3.56	-	-	- 4.26
IL8RB	−2.96	-	-	-	2.73	-	3.42	-

To examine the effect of Vpr mutation on differential gene expression in MDMs, HIV-1∆Vpr-infected culture was compared with mock-infected cultures (Table 2). Comparative analyses indicate that IL-1α, IL-1β, IL-8, TNF-α, C3 and BCL6 were upregulated during early time points (1–4 days) and were either downregulated or did not show significant difference during later infection phase (5–20 days post infection). Less infectivity of HIV-1∆Vpr virus in MDMs was supported by Table 2 because the initial differences observed on the first day of culture are not carried through at further time points although expression of few cytokines such as TNF-α, IL-5 and IL-10 was sporadically regulated during the infection phase.

**Table 2:** Differentially regulated inflammatory genes in MDMs infected with HIV-1 ∆Vpr compared to mock/uninfected (N = 6)

**Genes**	**Day1**	**Day2**	**Day3**	**Day4**	**Day8**	**Day12**	**Day16**	**Day20**
CD40LG	-	-	-	-	2.68	7.36	-	-
IFNA2	−2.95	-	−2.24	-	-	9.72	-	-
IL10	2.11	-	-	-	-	-	-	-
IL13	-	-	-	−2.18	−2.01	14.11	-	-
IL17C	-	-	-	-	-	3.29	-	-
IL1A	7.73	7.74	2.088	-	-	-	-	-
**IL1B**	**71.73**	**50.57**	**10.30**	**2.75**	**−2.17**	**-**	**-**	**-**
IL1F10	-	-	-	-	-	5.34	-	3.79
IL22	3.44	-	-	-	−2.63	4.96	−3.70	2.30
IL5	-	-	-	-	-	5.31	2.49	-
**IL8**	**23.79**	**16.27**	**6.30**	**2.61**	**-**	**-**	**-**	**-**
IL9	-	−2.74	-	-	-	5.32	-	−5.33
LTA	-	-	2.53	-	-	3.82	−3.04	2.88
TNF	3.86	2.46	-	-	-	-	-	2.6
IL10RA	-	2.45	-	−2.02	-	-	-	-
IL10RB	-	-	-	-	-	-	-	−2.82
IL9R	-	-	−2.14	2.95	-	5.76	-	2.84
IL13RA1	-	-	-	-	-	-	-	-
IL5RA	-	-	−2.71	-	−3.76	10.24	2.15	5.06
BCL6	3.24	4.27	2.26	2.67	-	-	-	2.40
C3	8.51	9.88	5.38	2.22	−2.04	−10.82	−4.36	-
C4A	-	-	-	-	-	-	-	2.34
CEBPB	-	-	-	-	-	-	-	6.26
CRP	-	-		-	-	13.47	−4.63	-
IL1RN	-	-	-	−2.31	-	-	-	-
IL8RB	−2.96	-	-	-	-	-	-	-
LTB4R	-	-	-	-	2.35	-	-	-
TOLLIP	−2.01	-		-	-	-	-	2.29

To delineate the specific effect of Vpr mutation in presence of other viral proteins in context of HIV-1^wt^, cytokine array results were compared between HIV-1^wt^ and HIV-1∆Vpr-infected MDMs (Table 3). Absence of Vpr downregulated several proinflammatory molecules such as IL-1α, IL-1β, IL-8 compared to HIV-1^wt^-infected culture in infection phase, suggest that Vpr could have a specific effect in activating proinflammatory factors, either directly or through enhanced viral replication. IL-1β and IL-8 were downregulated 4 days post infection and remained low in the absence of Vpr.

**Table 3:** **Differentially regulated inflammatory genes in MDMs infected with HIV-1 ∆Vpr compared to HIV-1**^**wt**^**(N = 6)**

**Genes**	**Day1**	**Day2**	**Day3**	**Day4**	**Day8**	**Day12**	**Day16**	**Day20**
CD40LG	-	-	-	-	-	-	-	2.37
IFNA2	-	-	-	-	-	3.05	-	-
IL10	-	-	-	-	-	-	-	−2.94
IL13	-	-	-	-	-	4.20	-	-
IL1A	-	-	-	-	-	−2.015	−2.12	−2.53
**IL1B**	**-**	**-**	**2.135**	**-**	**−3.149**	**−4.84**	**−4.76**	**−4.19**
ILF10	-	-	-	-	-	2.75	-	-
IL22	-	-	-	-	-	4.67	−3.21	-
IL36A	-	-	-	-	-	3.55	-	-
IL36B	-	-	-	-	-	3.228	-	-
IL36G	-	-	2.09	-	**2.004**	-	-	−2.27
IL5	-	-	-	-	-	**-**	**-**	−2.05
**IL8**	**-**	**-**	**-**	**-**	**−2.62**	**- 2.62**	**−3.64**	**−3.27**
IL9	-	--	-	-	-	-	-	2.12
LTA	-	-	-	-	-	-	−2.24	-
TNF	-	-	-	-	-	−2.41	-	**-**
IL1RN	-	-	-	-	-	-	-	**2.35**
IL5RA	-	-	-	-	-	6.18	-	**3.44**
IL9R	-	-	-	2.661	-	5.05	-	-

To determine the most significantly regulated proinflammatory genes, the gene array data were reanalyzed including all time points. Table 4 shows the proinflammatory genes that were differentially regulated in HIV-1∆Vpr-infected MDMs compared to HIV-1^wt^ combining all time points. Results indicate that Vpr mutant virus has a significant effect on downregulation of proinflammatory IL-1β and IL-8 in MDMs compared to HIV-1^wt^.

**Table 4:** **Differential expression of inflammatory factors in HIV-1∆Vpr-infected MDMs compared to HIV-1**^**wt**^-infecte**d (N = 6) including all time points**

**Gene symbol**	**Fold change**	**p-value**
IL5RA	2.091	0.269
XCR1	2.046	0.974
**IL1β**	**−4.178**	**0.033**
**IL8**	**−2.997**	**0.036**

**Figure 2:**
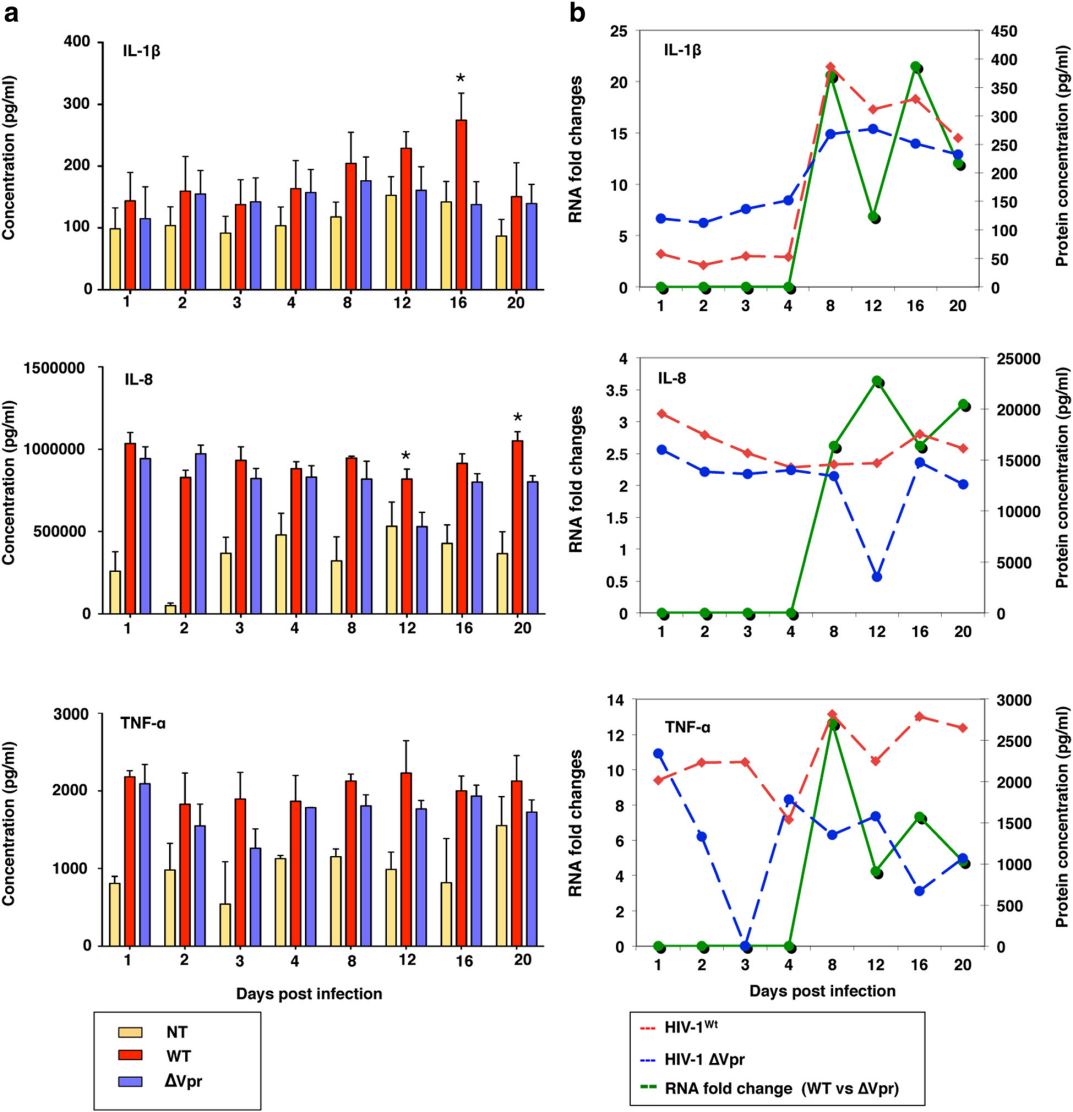
**HIV-1∆Vpr produces less proinflammatory IL-1β, IL-8 and TNF-α in MDMs than HIV-1**^**wt**^**.** MDMs were infected with HIV-1^wt^ or HIV-1∆Vpr virus at an MOI of 0.1 or left untreated for 1, 2, 3, 4, 8, 12, 16 and 20 days. **(a)** Expression of IL-1β, IL-8 and TNF-α in protein levels in supernatants of HIV-1^wt^, HIV-1∆Vpr and mock-infected (NT) MDMs collected at indicated time points was monitored by ELISA (N = 6). Absence of Vpr reduced the production of proinflammatory cytokines. The results presented are the mean concentration of cytokines ± SEM. *P < 0.05 (two tailed student’s *t*-test) compared to HIV-1∆Vpr-infected cultures. **(b)** qRT-PCR was carried out with the cell lysates as mentioned in Methods (N = 6). RNA fold changes were compared with ELISA data for each individual experiment. The expression of cytokines in protein levels (HIV-1^wt^; red lines and HIV-1∆Vpr; blue lines) was correlated with the respective RNA transcripts (fold changes in HIV-1^wt^ compared to HIV-1∆Vpr; green lines). Figure represents one of six independent experiments.

To further examine whether the differences noted with proinflammatory cytokines at the transcriptional level were also present at the protein levels, the supernatants from HIV-1^wt^, HIV-1∆Vpr and mock-treated MDMs from different time points were analyzed for IL-1β, IL-8 and TNF-α by ELISA. TNF-α was included for this study because it has already been indicated as a known proinflammatory marker [14]. Consistent with the data on transcript levels, absence of Vpr also reduced the release of IL-1β, IL-8 and TNF-α compared to HIV-1^wt^-infected MDMs. The concentrations of IL-1β and IL-8 were significantly (*P* = 0.045 for IL-1β at day 16; *P =* 0.0257 and *P =* 0.010 for IL-8 at day 12 and 20 respectively) higher in wild type-infected compared with ∆Vpr-infected culture at infection phase (Figure 2A). The increase and/or decrease in protein levels were directly correlated with the RNA transcript levels. Figure 2B represents one of six individual experiments demonstrating the correlation. For instance, IL-1β and TNF-α showed a decrease on day 8 both at the protein and transcript levels followed by a reduction on day 12 compared to HIV-1^wt^-infected culture.

### Absence of Vpr reduces activation of p38 and SAPK/JNK and not ERK1/2 in MDMs

**Figure 3:**
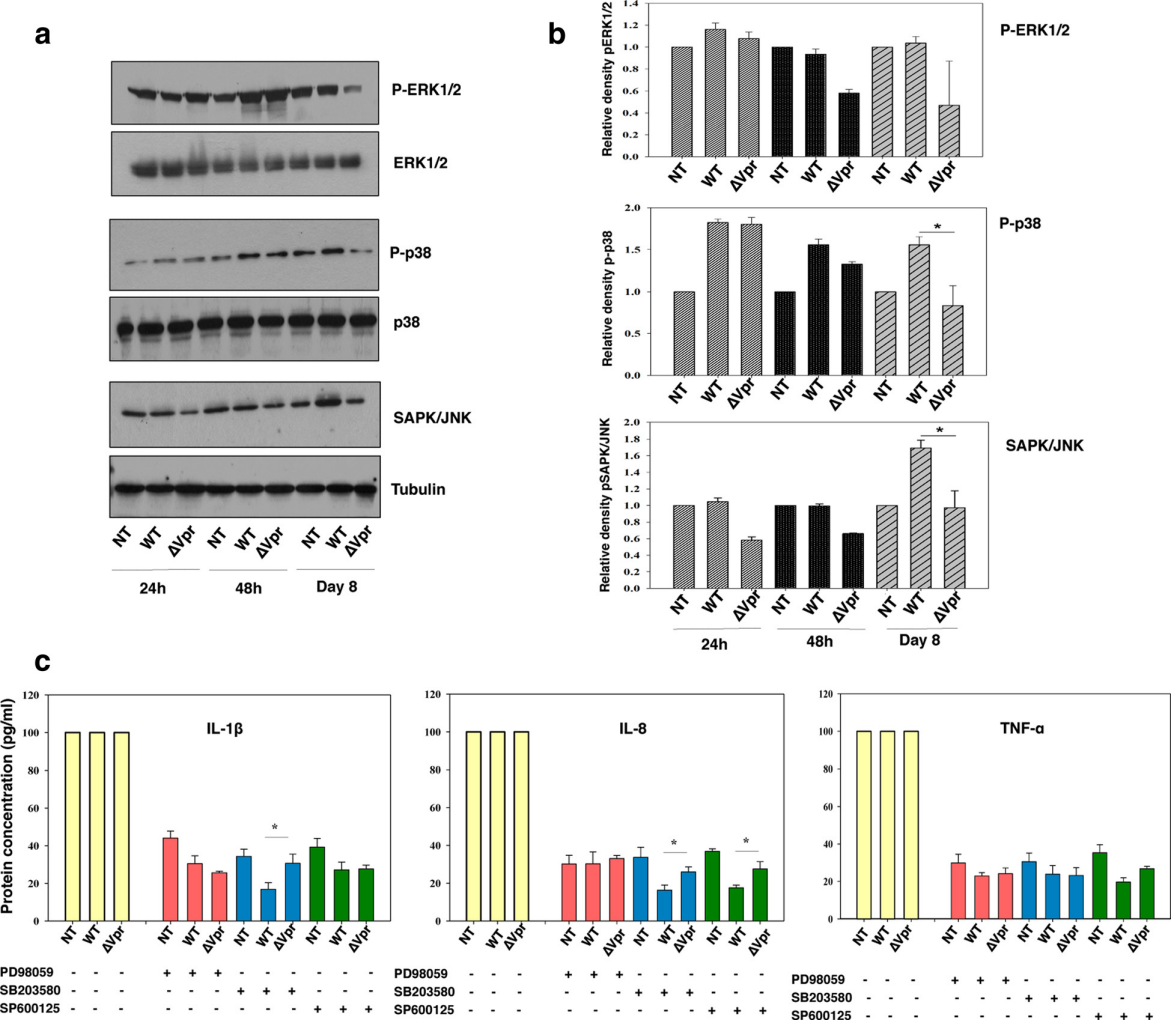
**HIV-1∆Vpr does not activate p38 and SAPK/JNK in MDMs as HIV-1**^**wt**^**.** MDMs infected with HIV-1^wt^, HIV-1∆Vpr or mock (NT) were activated with 1 μg/ml of LPS for 4 hours and the cells were harvested at 6, 12, 24, 48 hours, 4, 8, 12 days. **(a)** Cells were lysed and 50 μg of protein of each sample was analyzed by western blot using antibodies against the active and total form of ERK1/2, p38 and antibodies against SAPK/JNK and tubulin. **(b)** Relative band intensities of phosphorylated products were normalized with total proteins. Densitometrical quantification of western blot data represents the ± SEM of three independent observations. *P < 0.05 (two tailed student’s *t*-test) compared to HIV-1∆Vpr-infected cultures. **(c)** MDMs were preincubated with PD98059 (10 μM), SB203580 (10 μM) and SP600125 (10 μM) for 2 hours and then infected with HIV-1^wt^ or HIV-1∆Vpr virus at an MOI of 0.1 or mock. After 48 hours of infection IL-1β, IL-8 and TNF-α ELISA were performed. MDMs without MAPK inhibitors were considered to be the control and were reused to normalize other results. Each sample was run in triplicates, the results are ± SEM of the concentration of the cytokines and *P < 0.05 compared with HIV-1 ∆Vpr treated.

MAPK signaling cascade is known to play a role in the production of cytokine/chemokine by macrophages/microglia and astrocytes and hence activate immune response in the host cells [19,32,33]. Therefore, next we examined whether deletion of Vpr could modulate phosphorylation of the three most important members of MAPK family including ERK1/2, p38 and SAPK/JNK that are known to regulate proinflammatory cytokine production. Phosphorylation of ERK1/2, p38 and SAPK/JNK was assessed by western blot from 6 hours to 20 days post infection after activating the MDMs with LPS and the bands were quantified by densitometry (Figure 3A, B). Results indicate an increase in phosphorylation of p38, SAPK/JNK and ERK1/2 at 6 hours post infection in both HIV-1^wt^ and HIV-1∆Vpr-infected MDMs compared to control (data not shown). This increase was followed by a decrease at 12, 24 and 36 hours. Phosphorylation of MAPKs at 6 hours may be the consequence of gp120 binding and Vpr may not have specific effect at this stage. The difference of phosphorylation levels of p38 and SAPK/JNK between HIV-1^wt^ and HIV-1∆Vpr-infected MDMs was first observed at 48 hours post infection and was maintained up to day 8. Phosphorylation of SAPK/JNK was most pronounced at day 8. However, ERK1/2 showed no change in phosphorylation between HIV-1^wt^ and HIV-1∆Vpr-infected MDMs over a period of 20 days except the initial exposure (6 hours post infection).

To confirm the involvement of p38 and SAPK/JNK signaling molecules and to cross check the effect of ERK1/2 on IL-1β, IL-8 and TNF-α upregulation, MDMs were pretreated with SB203580, SP600125 and PD98059 prior to infection with HIV-1^wt^ or HIV-1∆Vpr or mock. Supernatants were collected 48 hours post infection and the concentrations of TNF-α, IL-1β and IL-8 by ELISA were measured (Figure 3C) in multiple donors (N = 3). MAPK inhibitors reduced the levels of all tested proinflammatory factors in all infected MDM supernatants compared to control MDMs. Blocking of ERK1/2 pathway with PD98059 showed reduction in IL-1β, IL-8 and TNF-α levels in both infected and uninfected MDM supernatants. However, SB203580, that blocks p38 pathway, significantly suppressed the production of IL-1β as well as IL-8 in HIV-1^wt^-infected MDMs compared to HIV-1∆Vpr-infected culture suggesting that activation of p38 pathway is involved in production of IL-1β and IL-8 in MDMs. However, SB203580 did not exhibit any specific difference in TNF-α level between HIV-1^wt^ and HIV-1∆Vpr virus-infected MDM cultures indicating that Vpr-induced TNF-α production could be mediated by other pathways. Pretreatment of MDM culture with SP600125, a selective inhibitor of JNK, specifically reduced the HIV-1^wt^-infected MDM-derived IL-8 production compared to mock treated or HIV-1∆Vpr-infected culture suggesting the association of SAPK/JNK pathway with IL-8 production in MDMs. No specific differential expression pattern was observed for IL-1β and TNF-α with SP600125 in presence or absence of Vpr. These results suggest that p38 and SAPK/JNK but not ERK1/2 are involved in IL-1β and IL-8 production. HIV-1∆Vpr activates these MAPKs to a less extent and hence induces less production of proinflammatory cytokines compared to HIV-1^wt^.

### Absence of Vpr reduces neuronal death in part through proinflammatory cytokines

**Figure 4:**
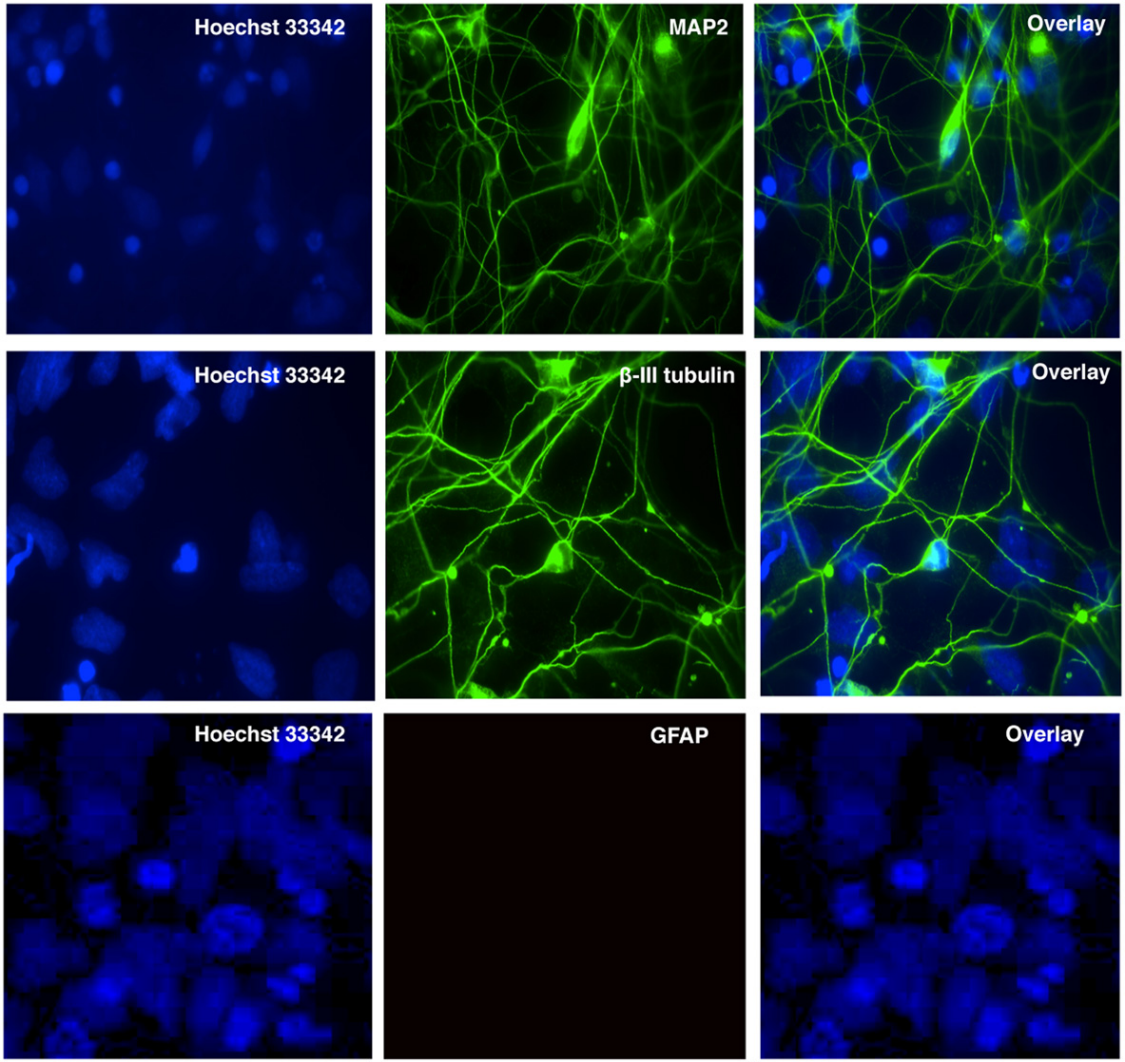
**Differentiation of NP cells to primary neurons.** Differentiation of the NP cells to primary neurons was performed for 2 weeks. Neuronal differentiation was confirmed by immunostaining. Neurons were fixed, permeabilized and stained with neuronal marker MAP2 (green) and β-III tubulin (green) or astrocyte specific marker GFAP (red). Nuclei were stained with Hoechst 33342 (blue). The figures are representatives of one of three independent experiments.

To obtain primary neurons for toxicity studies, NP cells were differentiated as described in Methods and confirmed by immunostaining using specific markers. One week post-differentiation the cells exhibited neuronal phenotype followed by increased expression of neuronal markers, MAP2 or β-III tubulin. Expression of MAP2 or β-III tubulin positive cell population increased to approximately 80% 2 weeks post differentiation (Figure 4). Astrocyte contamination was verified by immunostaining the cells using antibody against GFAP and results indicate absence of astrocytes in our cultures.

**Figure 5:**
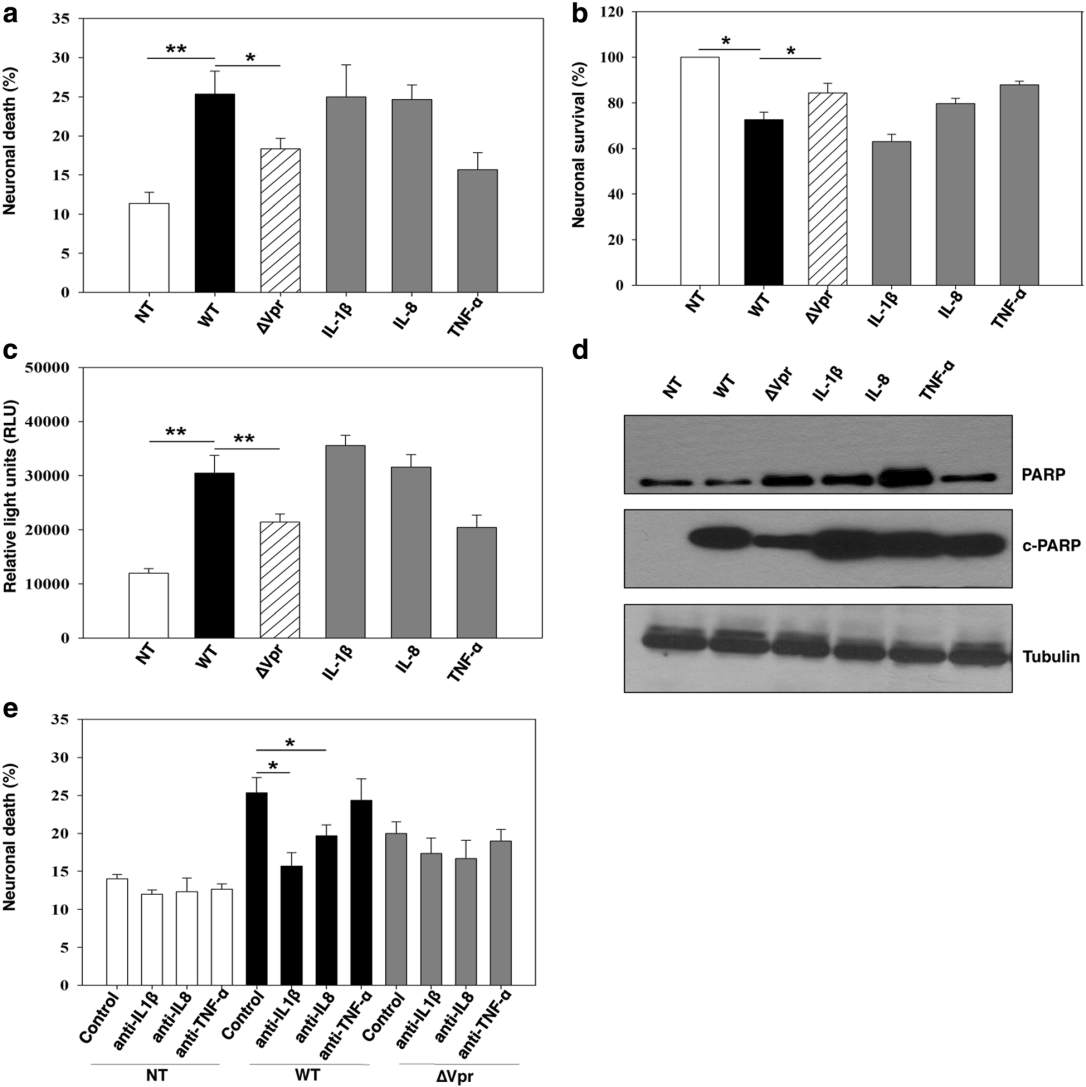
**HIV-1-induced neuronal death is mediated in part through IL-1β and IL-8 and not TNF-α present in supernatants of HIV-1**^**wt**^ **virus-infected MDMs.** Primary neurons exposed to HIV-1^wt^, HIV-1∆Vpr and mock-infected (NT) MDM supernatants (10% v/v) as well as recombinant IL-1β (10 ng/ml), IL-8 (10 ng/ml) and TNF-α (100 ng/ml) for 24 – 48 hours were analyzed for neuronal apoptosis by **(a)** Annexin-V FITC staining, nuclei of the cells were stained with Hoechst 33342 and measured the neuronal death (%); **(b)** MTT assay, absorbance was measured and cell viability was normalized with negative controls. HIV-1^wt^-infected MDM supernatants were more neurotoxic compared to HIV-1∆Vpr culture. **(c)** The cells were lysed in Caspase-Glo**®** 3/7 substrate and protease activity was measured as relative light units (RLU). **(d)** Neurons were lysed and 50 μg of protein for each sample was analyzed by western blot using specific antibodies for cleaved and total PARP. **(e)** Neurons exposed to supernatants from HIV-1^wt^, HIV-1∆Vpr and mock-infected MDM (NT) were treated with and without anti-IL-1β, anti-IL-8 and anti-TNF-α and neurotoxicity was analyzed after 24–48 hours by Annexin-V staining. Results are the ± SEM of three individual experiments; *P < 0.05, **P < 0.01 in HIV-1^wt^ compared with control or HIV-1 ∆Vpr treated.

Infected target cells in CNS compartment are known to dysregulate neuronal function and survival through proinflammatory factors [9,34,35]. To investigate the role of proinflammatory factors in inducing neuronal death, primary neurons were exposed to different amounts of culture supernatants from HIV-1^wt^, HIV-1∆Vpr and mock-infected MDMs (5%, 10%, 20% v/v in culture media). Supernatants from day 8 and day 12 were used, as IL-1β, IL-8 and TNF-α concentrations were highest at these time points. Neuronal death (%) was assessed 24 to 48 hours post treatment by Annexin-V staining. Recombinant proteins, rhIL-1β, rhIL-8 and rhTNF-α were used in parallel as positive controls. When neurons were exposed to different concentrations of MDM supernatants no difference in cell viability was observed at a concentration of 5% (v/v) exposure, whereas 10% (v/v) supernatant induced differential cell death and this effect tapered off with increasing concentration (data not shown). Hence all the experiments were performed with 10% MDM supernatants to assess neurotoxicity. Supernatant from mock-treated neurons exhibited approximately 11% cell death as basal level apoptosis, whereas neuronal culture exposed to HIV-1^wt^ or HIV-1∆Vpr-infected supernatant, exhibited approximately 25% (*P =* 0.0078) and approximately 17%, (*P =* 0.0349) cell death respectively. HIV-1∆Vpr-infected MDM supernatant resulted in significantly (*P =* 0.0481) lower neuronal death compared to HIV-1^wt^. Interestingly, the recombinant proteins rhIL-1β (approximately 27%) and rhIL-8 (approximately 24%) and not rhTNF-α (approximately 14%) exhibited similar significant cell death (Figure 5A).

To confirm this result, neuronal survival was also examined by MTT assay, which exhibited significantly reduced neuronal survival with HIV-1^wt^ (73%; *P =* 0.0139) but not with HIV-1∆Vpr (84%; *P =* 0.0669)-infected MDM supernatant compared to control. HIV-1∆Vpr-infected MDM supernatant further reduced the neurotoxic effect of the virus significantly (*P =* 0.0439) compared to HIV-1^wt^ (Figure 5B). Treatment of neurons with recombinant proteins, rhIL-1β, rhIL-8 and rhTNF-α exhibited 63%, 78% and 87% cell survival respectively, confirming their role in neuronal apoptosis.

Induction of apoptosis via death receptors activates initiator caspases, which in turn activate effector caspases 3 and 7. To investigate whether caspase 3/7 pathway is involved in inducing neuronal apoptosis neurons were pretreated with MDM supernatants (day 8 and 12) or recombinant proteins, for 24 hours and Caspase-Glo® 3/7 assay was performed. Exposure of neurons to HIV-1^wt^-infected MDM supernatants significantly increased caspase 3/7 activity compared to untreated or HIV-1∆Vpr-infected MDM supernatant (Figure 5C). Compared to mock-infected supernatant, HIV-1∆Vpr virus-infected supernatant exposure induced approximately 2-fold higher caspase 3/7 activity, whereas, HIV-1^wt^ virus-infected supernatant showed approximately 3-fold increased caspase 3/7 activity in neurons. Activation of caspase 3/7 in neuronal culture treated with recombinant proteins confirmed their role in neuronal apoptosis. Although rhIL-1β induced the highest activation (approximately 3-fold), rhIL-8 (approximately 2.5-fold) also significantly enhanced the caspase 3/7 levels in neurons. Compared to control, caspase 3/7 activity caused by rhTNF-α was <2-fold higher, which was much less compared to other recombinant proteins.

Furthermore, impaired activation of caspase 3/7 was also reflected in PARP expression in neurons. PARP, catalyzed by cleaved caspase 3 and 7, is the end product of caspase cascade and functions as a DNA-binding enzyme that detects DNA strand break [36,37]. Western blot results further confirmed that absence of Vpr induced a decreased level of cleaved PARP (c-PARP) in HIV-1∆Vpr-infected MDM supernatants-treated neurons compared to its HIV-1^wt^ counterpart (Figure 5D). Similarly, recombinant cytokines showed increased c-PARP signals in neuronal culture confirming their association with neuronal apoptosis. This result indicates that IL-1β, IL-8 and TNF-α might play a role either directly or by networking with some other downstream factors that in turn can activate neuronal death through caspase pathway.

### Neutralization of IL-1β and IL-8 protects neuronal death

To confirm that the neuroprotective effect of Vpr-deleted virus is mediated through proinflammatory factors released by MDMs, neurons were exposed to HIV-1^wt^, HIV-1∆Vpr and mock-infected MDM supernatants with or without IL-1β, IL-8 and TNF-α-neutralizing antibodies or isotype controls for 24 hours and neuronal apoptosis was determined by Annexin-V staining. Results showed that pre or post incubation of neurons with anti-IL-1β and anti-IL-8 antibodies restored 38% and 22% neuronal death in HIV-1^wt^ MDM supernatant-treated cultures, respectively whereas anti-TNF-α antibody had no significant effect (4%) in protecting HIV-1^wt^ MDM supernatant-induced cell death (Figure 5E). Interestingly, in HIV-1∆Vpr-infected MDM culture, although neutralizing antibodies also reduced neuronal death the effects were not significant. Collectively, these results confirm the role of Vpr-mediated indirect effect on neuronal survival via proinflammatory cytokines.

## Discussion

Neuroinflammation in the context of viral infections including HIV-1 could result from the following scenarios. First, the infiltration of infected monocytes/macrophages and lymphocytes from the periphery into CNS compartment; second, the release of viral and cellular factors by the infiltrated cells; third, the infection of resident macrophages/microglia by HIV-1 entering CNS or through virus released from the infiltrated cells. The infiltrated monocytes and lymphocytes are the key players of proinflammatory cytokines production [38,39]. The infected target cells are also known to secrete viral proteins including gp120, Tat, and Vpr, which are known to alter proinflammatory milieu in brain [24,40,41]. The role of gp120 and Tat in modulating proinflammatory cytokines and hence the effect on neurodegeneration has been studied extensively [16,42,43]. Few studies have also documented HIV-1 Vpr-mediated neuropathogenesis [24,44], however, effect of Vpr on neurotoxicity through proinflammatory cytokines remains undefined. HIV-1 Vpr has several features that may facilitate its role as a player in neuropathogenesis. Vpr, as a late viral protein synthesized in the infected cells, is released from the infected cells and is also taken up by nearby cells. Hence, the ability to cause damage is not confined to only virus-infected cells. Another interesting feature is that Vpr is also incorporated into the virus particles. This enables Vpr to be transferred to cells upon infection by the virus. It should be noted that virus particles, both in the infected individual and in cell culture, comprise a high proportion of non-infectious in comparison to infectious particles. Although non-infectious virus particles are replication defective, they are still capable of transferring viral proteins such as Vpr into target cells. This shows that Vpr can cause damage through multiple avenues. In an effort to analyze the effect, previous studies focused on using specific viral proteins (purified recombinant proteins) in the absence of other viral proteins. Although these studies provided some insight, unfortunately the concentrations used are in the non-physiological range. This is the basis for our studies aimed at investigating the indirect effect of Vpr deletion on protection from neuronal damage (in comparison to HIV-1^wt^) through proinflammatory cytokine network using replication competent HIV-1^wt^ and HIV-1∆Vpr-infected MDMs.

Expression of proinflammatory cytokines was upregulated in HIV-1^wt^-infected MDMs compared to controls at the transcriptional level. In Vpr-deleted virus-infected culture, most of the proinflammatory cytokine genes were upregulated only in the first four days when compared with mock. The increased expression of these cytokines immediately upon infection and not at the infection phase could be an immediate effect of HIV-1 envelope protein binding to cell surface receptors or may be indicative of less HIV-1∆Vpr virus infectivity in MDMs at later time points. Compared to HIV-1^wt^, HIV-1 ∆Vpr-infected MDMs exhibited downregulation of IL-1β and IL-8 four days post infection and maintained the low level up to day 20. Lower expression of TNF-α was also observed at protein level in HIV-1∆Vpr culture compared to HIV-1^wt^, although no significant difference was found in transcriptional level.

Deletion of Vpr-suppressed IL-1β, IL-8 and TNF-α production in MDMs suggests that Vpr plays a role in the production of these cytokines. However, it is not clear whether Vpr specifically regulates these cytokines directly through transcriptional regulation or by enhanced MDM infection [45]. It has been shown that Vpr either as a virion-associated molecule or as a free protein is known to act as a transcriptional regulator [46-48]. Our preliminary bioinformatics analyses (data not shown) indicate the presence of several common transcription factor-binding sites present in IL-8 and IL-1β promoters suggesting that they could be potentially involved in increased expression of these genes. However, many of these sites are also shared by other cytokines such as TNF-α (four common in all three) suggesting that Vpr might utilize these transcription factors to upregulate these cytokines in an additive manner. In-depth chip-based analyses are required to address these questions, which is beyond the scope of this manuscript. Alternatively, the coincidence of decreased HIV-1 replication with decrease of IL-1β, IL-8 and TNF-α levels in HIV-1 ∆Vpr-infected MDMs with time indicates virus replication could also be one of the major determinants of increased expression of proinflammatory factors [45].

IL-1β and TNF-α have a broad range of similar and complex physiological effects including their ability to induce expression of a number of genes depending on the cell lineage. These two cytokines stimulate the production of chemotactic factors such as IL-8 in cells of CNS [49]. Hence, it is possible to speculate that IL-8 downregulation in HIV-1∆Vpr-infected MDMs may be a consequence of decreased levels of IL-1β and TNF-α compared to HIV-1^wt^-infected culture.

Expression of the proinflammatory factors in HIV-1 disease can be partly explained through the influence of HIV-1 on signaling pathways. Although HIV-1 interacts with several signaling molecules, in this study we focused on MAPK because these kinases are involved in many cellular activities including activation, proliferation, differentiation, survival and cytokine production [50-52]. Furthermore, signaling pathways controlling IL-1β, IL-8 and TNF-α gene expression have been linked to MAPK [53,54]. Several viral proteins such as gp120, Nef and Tat are known to interact with MAPK pathways [55-57]. Hence, we investigated if deletion of Vpr could suppress activation of MAPK pathways in MDMs; that could indicate indirectly the involvement of MAPKs in Vpr-induced differential regulation of proinflammatory cytokines. We observed decreased activation of p38 and SAPK/JNK in HIV-1∆Vpr compared to HIV-1^wt^-infected MDMs stimulated with LPS. Studies have shown that gp120 activates p38 during early phase of exposure/infection via chemokine and HIV-1 co-receptors binding [58]. However, it is unclear how phosphorylation p38 and SAPK/JNK are associated with the proinflammatory cytokine production in MDM. One explanation could be that activation of these signaling molecules may phosphorylate and/or translocate transcription factors that may activate promoters of IL-1β and IL-8 and upregulate gene expression. In the current study phosphorylation of ERK1/2 was not enhanced in response to infection, which is quite similar to an early study where the authors showed that Vpr induces cell cycle arrest through downregulation of ERK pathway rather than change in phosphorylation status [59].

Although HIV-1 does not infect neurons directly, the cytopathic effects on neurons are probably caused by macrophage/microglia-derived proinflammatory cytokines. The neurotoxic and proinflammatory cytokines implicated in HAND pathogenesis are IL-1β and TNF-α [12,14]. These factors have been reported to increase the permeability of blood–brain barrier and also over stimulate NMDA receptors, which cause lethal neuronal increase in Ca^2+^ levels [60]. IL-8 also could function as a mediator of neuronal death via its effects on release of neurotoxins such as matrix metalloproteinase as well as by induction of cell cycle and pro-apoptotic proteins [61]. A recent study reported that IL-8 levels in CSF of HAND patients are higher compared to HIV-1 seropositive patients without neurological disorders [19]. HIV-1 gp120 and Vpr-induced increase in IL-8 production in CNS is reported [62,63]. Suppression of production of these neurotoxic proinflammatory cytokines could be a possible way to reduce risk of neuronal apoptosis. In our study, deletion of Vpr caused significant reduction of proinflammatory cytokine IL-1β, IL-8 and TNF-α production, coinciding with the viral replication. This suggests that Vpr has both direct and indirect effects on the cytokine production. The exact mechanism of Vpr in neuropathogenesis is not prominent, although it is known to activate both intrinsic and extrinsic pathways. Soluble Vpr causes neuronal apoptosis, involving cytochrome *c* extravasation and p53 induction [44]. Our results on neuronal apoptosis and effector caspase activation upon exposure to HIV-1^wt^-infected MDM supernatants showed increased caspase-mediated neuronal death suggesting that soluble factors present in these cultures are responsible for neuronal death. This result is consistent with other studies, which also demonstrated increased caspase activation in presence of rVpr [44,64]. However, this does not delineate whether the soluble viral proteins and/or cellular factors present in MDM supernatant are responsible for neuronal damage. Results of partial restoration of neuronal death in presence of neutralizing antibodies against IL1β and IL-8 suggest that HIV-1 Vpr-induced IL-1β and IL-8 could possibly be the two important factors that affect neuronal apoptosis. Previous studies showed that administration of rhIL-1β enhances ischemic brain edema formation, size of the brain infarction, increases neuronal death, whereas, administration of anti–IL-1β reverses the neuronal death in rat model [65]. Compared to an HIV-1 positive patient without neurological disorder, HAND patients have increased levels of IL-8, the production of which is probably induced by IL-1β and TNF-α through MAPK pathways [19].

## Conclusion

Overall, our results demonstrate that HIV-1 Vpr plays an important role in MDM infection as well as production of proinflammatory and neurotoxic cytokines IL-1β and IL-8 by infected/exposed MDMs. These cytokines have already been reported to be associated with prevalence, frequency and severity of neurological disorders. Currently, different studies are in progress focusing on targeting cytokines as a therapeutic strategy for treatment of neurocognitive diseases. Deletion or alteration of Vpr reduces the production of proinflammatory cytokines and partially rescues neurons from cell death, thus targeting HIV-1 Vpr could be one possible approach to reduce the risk of HAND.

## Competing interests

The authors declare that they have no competing interests.

## Authors’ contribution

DG performed the experiments, analyzed the data and wrote the manuscript; PN performed the experiments and analyzed the data; CR generated the primary neurons; AS and GS wrote the manuscript; VA designed the study, analyzed the data and wrote the manuscript. All authors read and approved the final manuscript.
